# Bcl6/p53 expression, macrophages/mast cells infiltration and microvascular density in invasive breast carcinoma

**DOI:** 10.18632/oncotarget.25220

**Published:** 2018-04-27

**Authors:** Roberto Tamma, Simona Ruggieri, Tiziana Annese, Giovanni Simone, Anita Mangia, Serena Rega, Francesco A. Zito, Beatrice Nico, Domenico Ribatti

**Affiliations:** ^1^ Department of Basic Medical Sciences, Neurosciences and Sensory Organs, University of Bari Medical School, Bari, Italy; ^2^ National Cancer Institute “Giovanni Paolo II”, Bari, Italy; ^3^ Department of Pathology “San Paolo” Hospital, Bari, Italy

**Keywords:** Bcl6, breast cancer, macrophages, mast cells, p53

## Abstract

To better understand the breast cancer progression and therapeutic resistance is crucial deepen the molecular mechanisms related to regulation of cells behavior in the tumor microenvironment. Inappropriate expression or activation of transcription factors in tumor breast microenvironment can lead to the malignant behavior of breast cancer cells. Bcl6 is a transcriptional factor that may play a role in the pathogenesis of breast cancer. Moreover, cells surrounding tumor cells, including macrophages and mast cells play an important role during tumor progression enhancing angiogenesis. We have demonstrated: 1) An increase of the BCL6 translocation and Bcl6 positive cells in G3 degree of disease; 2) A reduction of the expression of p53 in G3 breast cancer samples as compared to G1/G2 specimens; 3) Macrophages CD68^+^ and CD163^+^ in interstitial and periglandular position, increase in G3 specimens as compared to G1/G2 and control samples; 4) Tryptase-positive mast cells in periglandular position are more numerous in G3 tumor specimens as compared to G1/G2 and control samples. Overall, these data confirm the important role played by epigenetic events, including BCL6 translocation, p53 expression, and microenvironment components, including macrophage and mast cell infiltration and microvascular density involved in the regulation of breast cancer progression.

## INTRODUCTION

Breast cancer remains one of the most controversial malignancy. Despite of the fact that conventional histopathology was completed with an accurate molecular stratification, additional factors influencing malignant progression exist [1].

Tumor microenvironment is emerging as a crucial aspect in the progression of tumors, including breast cancer. The initiation of breast cancer is due to transforming events in a single cell [1]. However, host microenvironment plays an important role in breast cancer tumorigenesis and tissue microenvironment changes during tumor formation and in the progression from normal mammary gland to ductal carcinoma in situ and finally to invasive ductal carcinoma [2]. Infiltration of lymphocytes, macrophages, mast cells, and neutrophils is a hallmark of tumor progression in breast cancer [3, 4]. Moreover, another factor involved in tumor progression in breast cancer is the transcription factor Bcl6, which is a gene promoter involved in p53 protein regulation in terms of suppression of its expression [5–8].

In this study, we have evaluated in a selected group of invasive breast carcinoma tumor specimens, Bcl6, p53 expression, apoptosis by TUNEL assay and caspase3 expression, macrophages, mast cells, and micro vessel density, to further clarify the crucial role played by epigenetic events and by the tumor microenvironment during breast cancer progression.

## RESULTS

### BCL6 translocation

**Figure 1:**
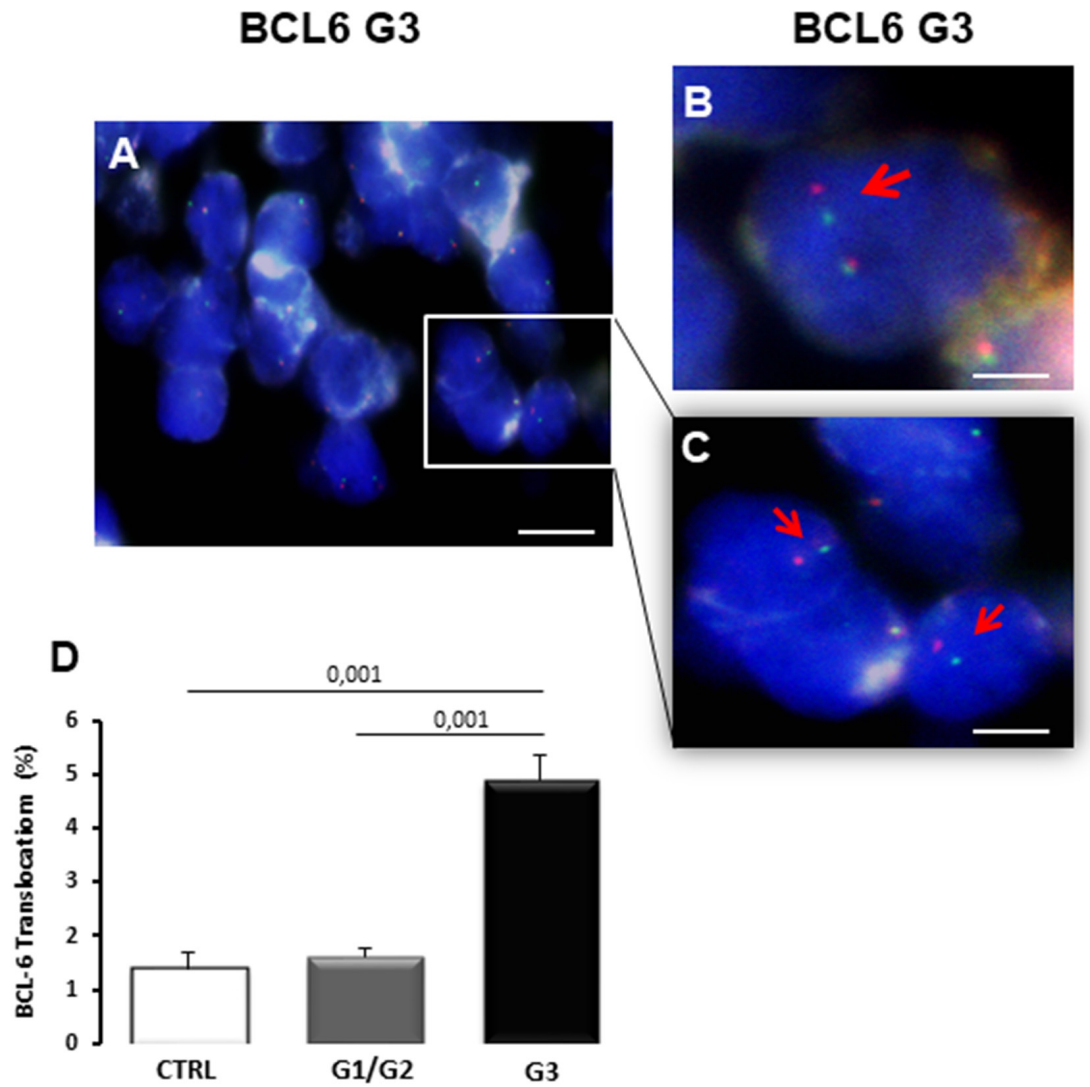
BCL6 translocation in bioptic samples of control (CTRL), G1/G2 and G3 breast cancer samples Interphase fluorescent *in situ* hybridization (FISH) analysis of G3 breast cancer samples shows BCL6 translocations as observed by separated FISH signal probes in same the nuclei **(A-C)**, indicated with red arrows (B, C). Interphase FISH quantitation **(D)** shows the increase of BCL6 translocations percentage in G3 samples. Scale bar: A 6 μm; B 2 μm; C 3,6 μm.

To investigate the BCL6 translocation in breast tumors with different degrees of malignancy, interphase FISH analysis on thin sections of the FFPE tumor samples, was performed. Interphase FISH analysis (Figure 1A) showed red and green separate fluorescent spots in the same nuclei of tumor tissues indicating BCL6 translocation (Figure 1B, 1C arrow). Interphase FISH analysis showed a significant increase of the BCL6 mutation in G3 degree of disease (Figure 1D) compared to healthy controls. In G1/G2 tumor samples, BCL6 mutation did not change as compared to the control. In G3 breast cancer, rearrangement of BCL6 showed a frequency of 4.9% ± SE 0.45%, *vs* a significant decrease to 1.62% ± SE 0.15% and 1.40% ± SE 0.29% in G1/G2 breast cancer and in control respectively (Figure 1D).

### Bcl6 expression

**Figure 2:**
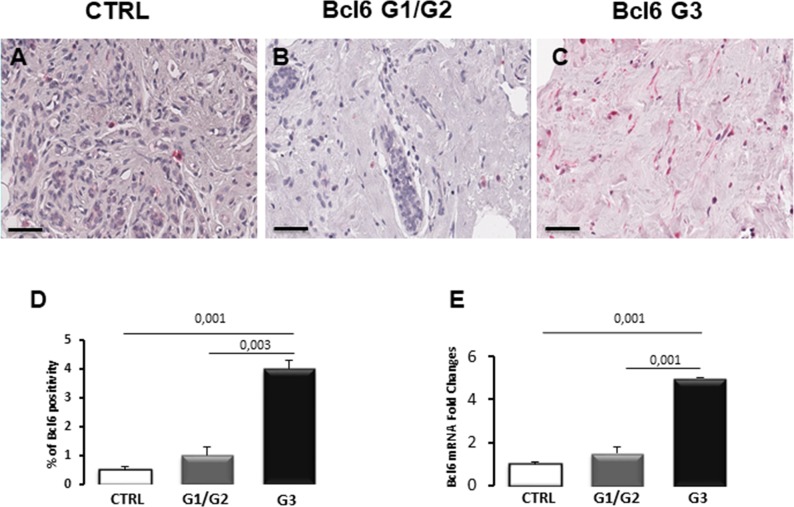
Immunohistochemical staining **(A-C)**, morphometric analysis **(D)** and messenger expression **(E)** of BCL6 in control (CTRL), G1/G2 and G3 breast tumor samples. The per cent of Bcl6 positive cells significantly increases in G3 tumor samples as compared to G1/G2 tumor samples and controls. Furthermore, Bcl6 mRNA expression significantly increases in G3 tumor samples as compared to G1/G2 tumor samples and controls. Scale bar: A-C 60 μm.

**Figure 3:**
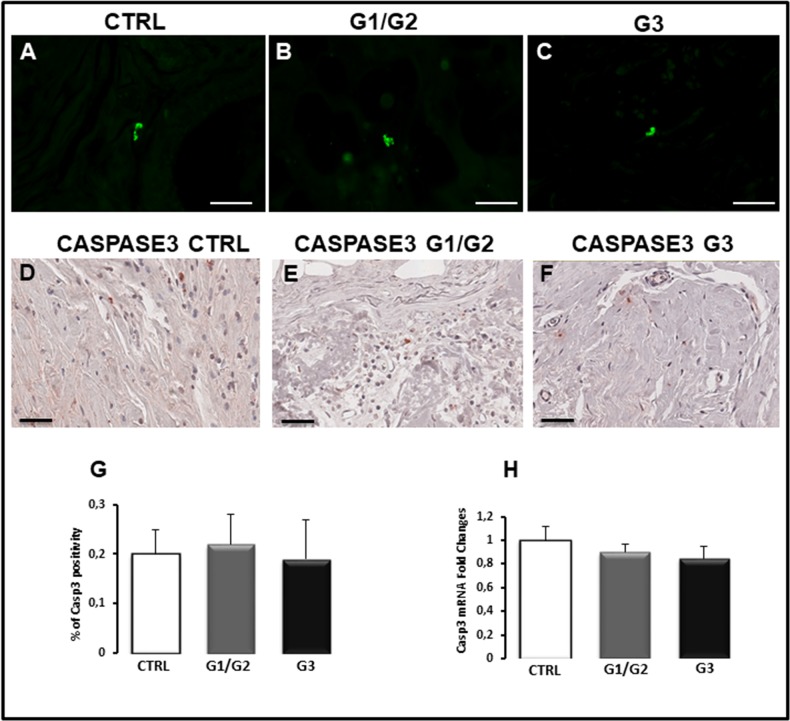
TUNEL assay **(A-C)**, immunohistochemical staining **(D-F)**, morphometric analysis **(G)** and messenger expression **(H)** of caspase-3 in control (CTRL), G1/G2 and G3 breast tumor samples. Apoptosis in no detectable by TUNEL assay (A-C), while the immunohistochemical staining (D-F), morphometric analysis (G) and messenger expression (H) don't show significant differences in caspase-3 expression between tumor specimens and controls. Scale bar: A-C 28 μm; D-F 60 μm.

To assess the Bcl6 expression pattern, immunohistochemistry and real time-PCR assay were performed. A low immunohistochemical expression of Bcl6 has been seen in control breast tissues (Figure 2A) as well as in G1/G2 breast cancer specimens (Figure 2B), while a strong increase of Bcl6 expression in G3 breast cancer specimens (Figure 2C) was detected. Morphometric analysis showed a significant difference (4.0% ± SE 0,3%) in Bcl6 expression in G3 breast cancer compared with G1/G2 breast cancer (1,1% ± SE 0,3%) and controls (0,50% ± SE 0,1%) (Figure 2D). Real time-PCR (Figure 3E), Bcl6 mRNA showed significant increase expression in G3 breast cancer (0.5 ± SE 0.05) compared with G1/G2 (1.5 ± SE 0.30) and control (1 ± SE 0.1) specimens. There was no significant difference between G1/G2 breast cancer and control specimens.

### TUNEL test, caspase-3 and p53 expression

**Figure 4:**
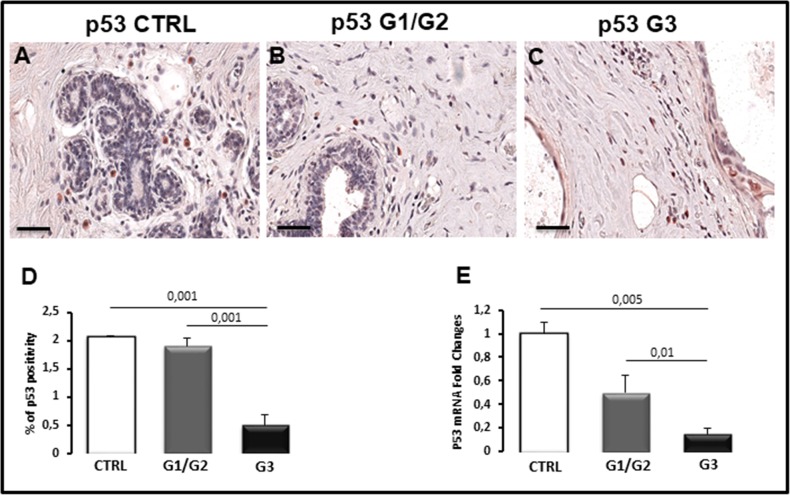
Immunohistochemical **(A-C)**, morphometric analysis **(D)** and messenger expression **(E)** of p53 in control (CTRL), G1/G2 and G3 breast tumor samples. The per cent of p53-positive cells (D) and the expression of p53 messenger (E) significantly decreases in G3 (C) tumor specimens as compared to G1/G2 (B) tumor specimens and controls (A). Scale bar: A-C 60 μm.

Apoptosis was studied in breast cancer specimens by TUNEL assay for measuring DNA strand breaks in individual cells and by the evaluation of caspase-3 and p53 expression. TUNEL assay revealed no apoptosis in all breast cancer samples (Figure 3B, 3C) as well as in control tissues (Figure 3A), and only few fluorescent apoptotic cells were detected in all analyzed sections (Figure 3A–3C). Caspase-3 immunohistochemical staining showed few labeled tumor cells in breast cancer sections (Figure 3E, 3F) as well as in control specimens (Figure 3D). Morphometric analysis confirmed a low percentage of caspase-3 positive cells in breast samples comparable to the control (Figure 3G), accordingly with the TUNEL test. The same result was obtained by Real Time-PCR (Figure 3H). The expression levels of caspase-3 mRNA in G1/G2 breast tumor samples (0.9 ± SE 0.07) as well as in G3 specimens (0.85 ± SE 0.1) were approximately comparable to control (1 ± SE 0.12). P53 protein, which plays a key role as apoptosis inducer, was also expressed at low levels in breast tumors as compared to the control (Figure 4). Microscopic observation and the quantitation analysis revealed that p53 expressing cells was significantly reduced (0.5% ± SE 0.18%) in G3 samples (Figure 4C, 4D) as compared to G1/G2 tumors (1.9% ± SE 0.15%) (Figure 4B, 4D) and controls (2.1± SE 0.03) (Figure 4A, 4D). Moreover, the significant decrease of p53 mRNA (Figure 4E), was detected in G3 breast cancers (0.15± SE 0.05) compared to G1/G2 tumor samples (0.50 ± SE 0.15) and control (1 ± SE 0.10) specimens. No significant difference between G1/G2 breast cancer and control specimens was detected. A correlation study on the relationship between p53 positivity and BCL6 translocation frequency was performed in all the G1, G2 and G3 patients. A negative correlation was found as assessed by Spearman correlation analysis (rho = −0.37, *p*<0.0015).

### TCGA data analysis

The research of P53 and BCL6 mutations in invasive breast carcinoma in the Cancer Genome Atlas (TCGA) portal showed that p53 presented 240 different mutations in 360 of 960 cases of invasive breast carcinomas, corresponding to 36.51% and BCL6 was mutated in 5 of 960 cases corresponding to 0.51% (Table 1).

**Table 1:** Association between BCL6 translocation frequency, p53 cell positivity, P53 mutations and clinic-pathological characteristics of the patients

	No. of patients	BCL6 Translocation		BCL6 mutation	P53 expression		P53 mutation	P53^*^ mutation
		positive nuclei	P-value	n (%)	positivity %	P-value	n (%)	n
**TCGA mutation (%)**	**960**			**5 (0,51%)**			**360 (36,51)**	
**Age (years)**								
≤45	28	2,5			1,5			
>45	42	2,5952	0,67		1,5	0,84		
**Tumor size (cm)**								
≤3	**45**	2,5333			1,3636			
>3	**25**	2,6	0,7		1,8	0,15		
**Histological Grade**								
G1	**30**	1,73			1,9667		3 (10)	
G2	**20**	1,45	0,32		1,8	0,59	1 (5)	
G3	**20**	**4,9**	**0,001**		**0,5**	**0,01**	7 (35)	1
**Estrogen Receptor**								
0 (%<10)	**0**							
1 (10 ≤%≤30)	**14**	1,5714			1,5714			
2 (30 ≤%≤60)	**19**	2,3684	0,21		3,3684	0,23		
3 (%60)	**37**	2,5135	0,32		2,5135	0,104		
**Progesterone Receptor**								
0 (%<10)	**13**	3			1,5			
1 (10 ≤%≤30)	**15**	1,8667	0,38		1,6	0,65		
2 (30 ≤%≤60)	**10**	2,3	0,74		1,4	0,97		
3 (%60)	**31**	2,875	0,9		1,4375	0,89		
^*^ DNA binding sites								

### Mutation screening of P53

The sequencing of the of P53 coding regions showed that 3 cases of 30 of G1 samples (10%), 1 case of 20 of G2 samples (5%) and 7 cases of 20 (35%) of G3 were mutated. Only 1 case of the P53 mutated in G3 presented the mutation in the DNA binding site. No mutations have been found in the Bcl6 binding regions region (Table 1).

### CD68, CD163, tryptase, and CD31 expression

**Figure 5:**
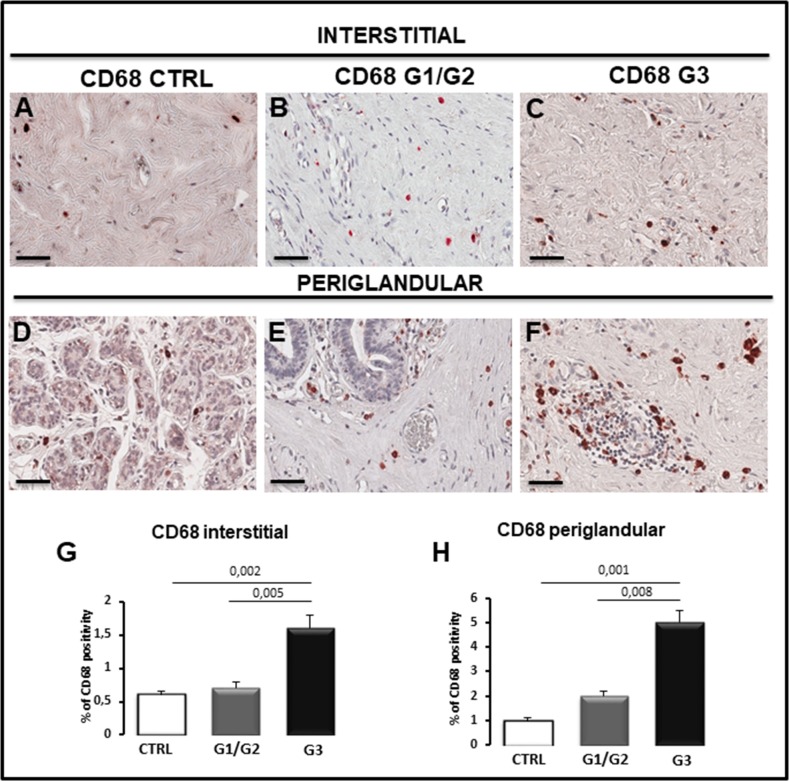
Immunohistochemical **(A-F)** and morphometric analysis **(G, H)** of CD68-positive macrophages in interstitial (A-C, G) and periglandular (D-F, H) position in control (CTRL), G1/G2 and G3 breast tumor samples. The per cent of CD68-positive cells significantly increases in G1/G2 and G3 tumor samples as compared to controls. Scale bar: A-F 60 μm.

**Figure 6:**
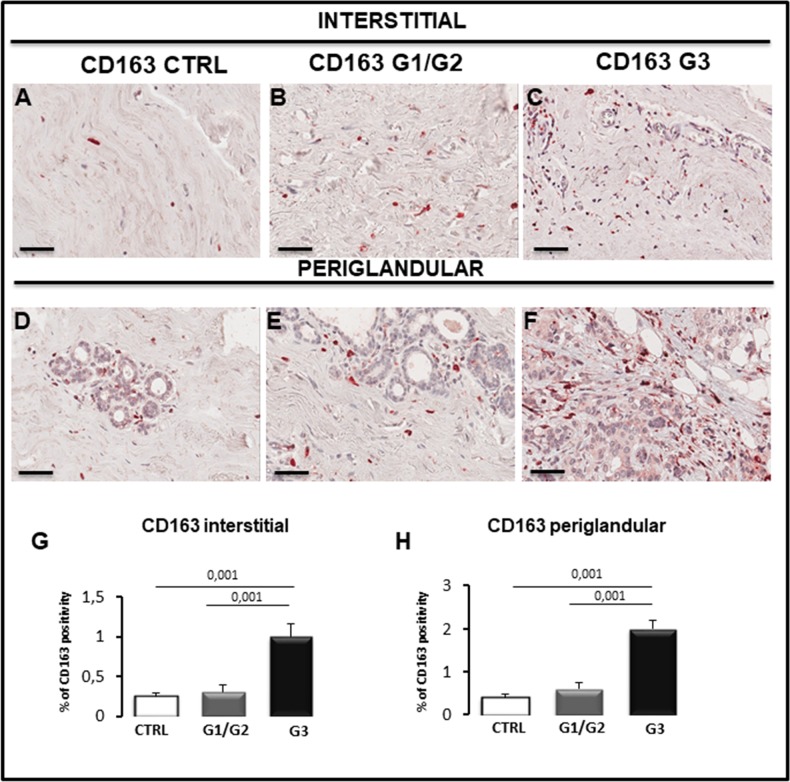
Immunohistochemical **(A-F)** and morphometric **(G, H)** analysis of CD163-positive macrophages in the interstitial (A-C, G) and periglandular (D-F, H) position in control (CTRL), G1/G2 and G3 breast tumor samples. The per cent of CD163-positive macrophages in both interstitial and periglandular position significantly increases in G3 tumor samples as compared to G1/G2 tumor samples and controls. Scale bar: A-F 60 μm.

**Figure 7:**
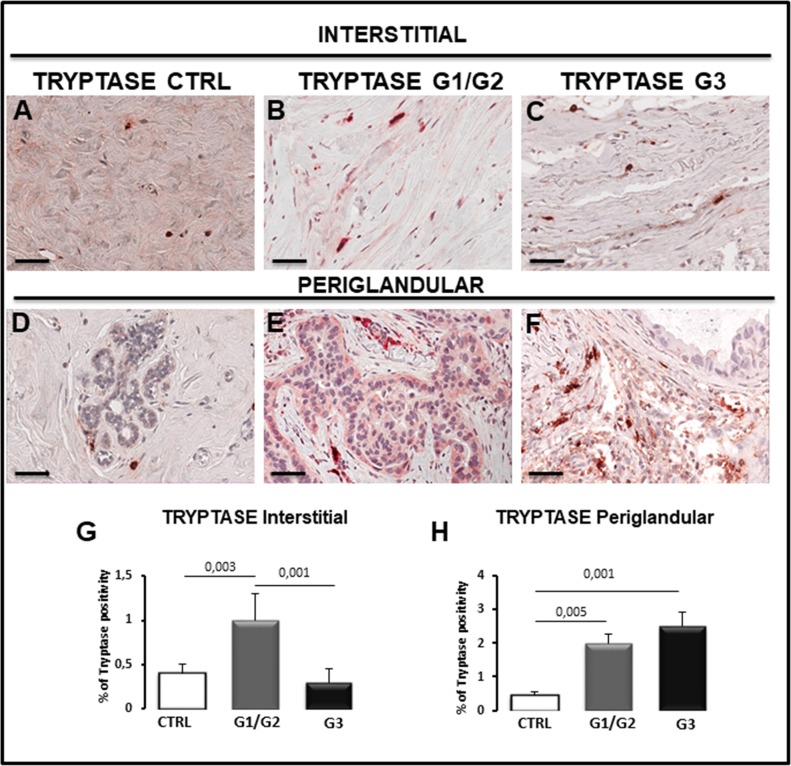
Immunohistochemical staining **(A-F)** and morphometric analysis **(G, H)** of tryptase-positive mast cells in the interstitial (A-C, G) and periglandular (D-F, H) position in control (CTRL), G1/G2 and G3 breast tumor samples. The per cent of tryptase-positive cells in interstitial position significantly increases in G1/G2 tumor samples as compared to controls and G3 tumor samples, while the percentage of tryptase-positive cells in periglandular position significantly increases in G1/G2 and G3 tumor samples as compared to controls. Scale bar: A-F 60 μm.

Breast G1/G2, G3 grade tumor and control specimens were immunostained for CD68, CD163, tryptase and CD31 to estimate total macrophages, M2-macrophages (Figure 5A–5H and Figure 6A–6H), mast cells (Figure 6A–6H), endothelial cells (Figure 7A–7H) in the interstitial spaces and in periglandular position.

As concerns CD68^+^ macrophages, their number in interstitial tissues increase significantly in G3 specimens (1.61% ± SE 0.2%) as compared to G1/G2 (0.7% ± SE 0.09%) and control samples (0.60% ± SE 0.06) (Figure 5A–5CA, 5G); moreover, a significant increase (5.0% ± SE 0.5%) of CD68^+^ cells in periglandular position was observed in G3 specimens as compared to the G1/G2 (2.0% ± SE 0.21%) and control samples (1.05% ± SE 0.10%) (Figure 5D–5FD, 5H). A correlation study on the relationship between CD68 and CD31 expression was performed. A positive correlation between CD68 and CD31 in G1/G2 samples in periglandular positions was found as assessed by Spearman correlation analysis (rho = 0.631, *p* < 0.03). We have also estimated the M2 subpopulation of macrophages, by using a specific antibody anti-CD163. Their number in interstitial tissues increase significantly in G3 samples (1 % ± SE 0.16%) as compared to G1/G2 (0.3% ± SE 0.1%) and controls (0.25% ± SE 0.04) (Figure 6A–6CA, 6G); moreover, a significant increase (2.1% ± SE 0.2%) of M2-macrophages was observed in periglandular position in G3 specimens as compared to the G1/G2 (0.6% ± SE 0.15%) and control samples (0.4% ± SE 0.07%) (Figure 6D–6FD, 6H). A correlation study on the relationship between CD163 and CD31 expression was also performed. A positive correlation between CD163 and CD31 in G1/G2 samples in periglandular positions was found as assessed by Spearman correlation analysis (rho = 0.531, *p* < 0.03).

As concerns tryptase-positive mast cells present in interstitial tissues, they were more numerous in G1/G2 specimens (1.0% ± SE 0.3%) as compared to G3 (0.30% ± SE 0.15%) and control (0.40% ± SE 0. 1%) specimens (Figure 7A–7CA, 7G), while tryptase-positive mast cells in periglandular positions were more numerous in G1/G2 (2.0% ± SE 0.25%) and G3 (2.5% ± SE 0.41%) as compared to the control (0.45% ± SE 0.1%) specimens (Figure 7D–7FD, 7H).

**Figure 8:**
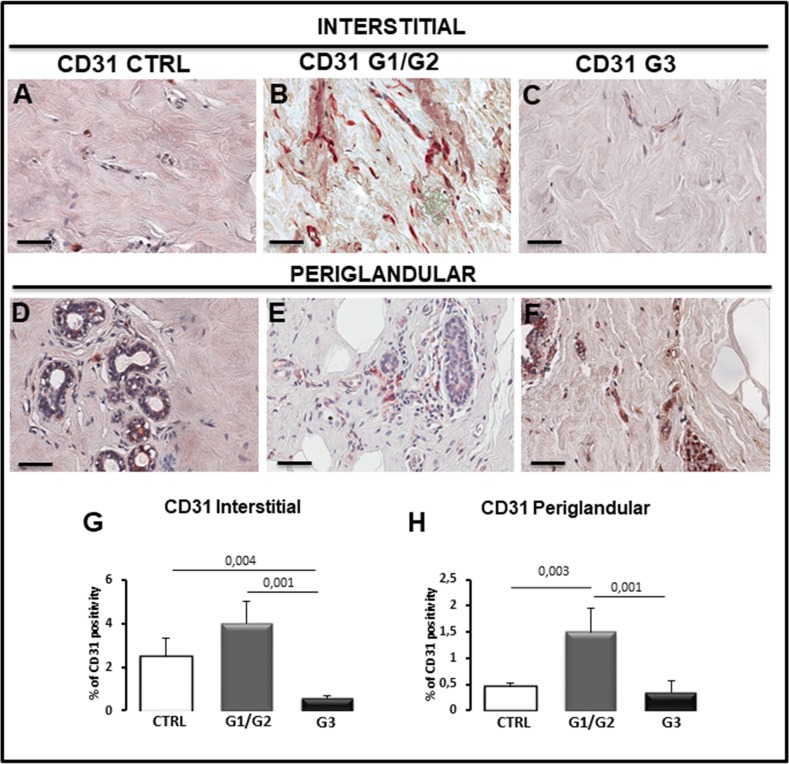
Immunohistochemical **(A-F)** and morphometric **(G, H)** analysis of CD31-positive microvessels in the interstitial (A-C, G) and periglandular (D-F, H) position in control (CTRL), G1/G2 and G3 breast tumor samples. The per cent of CD31-positive microvessels in both interstitial and periglandular position significantly increases in G1/G2 tumor samples as compared to G3 tumor samples and controls. Scale bar: A-F 60 μm.

As concerns CD31^+^ microvessels, they were increased in G1/G2 samples in both interstitial (Figure 8A–8CA, 8G) and periglandular positions (Figure 8D–8FD, 8H) as compared to G3 and control specimens. Moreover, the morphometric analysis showed that the interstitial tissue of G3 specimens contain less CD31 cells (0.6% ± SE 0.8%) as compared to all the samples (G1/G2: 4% ± SE 0.9%; Control: 2.5% ± SE 0.8%) (Figure 8G) whereas the G3 periglandular tissue (0.30% ± SE 0.02%) was reduced as compared to G1/G2 (1.51% ± SE 0.45%) but not as compared to control (0.45% ± SE 0.08%) (Figure 8H). A correlation study on the relationship between tryptase and CD31 expression was performed. A positive correlation between tryptase and CD31 in G1/G2 periglandular position (rho =0.681, *p*< 0.025) was found as assessed by Spearman correlation analysis.

## DISCUSSION

The proto-oncogene BCL6, located on chromosome 3, encodes the transcriptional repressor Bcl6 which is considered to be the master regulator of germinal center and is crucial for the development and function of B and T cells. Furthermore, Bcl6 is involved in the regulation of many cellular functions including cell proliferation and apoptosis. In breast cancer, Bcl6 prevents mammary epithelial cell differentiation, induces epithelial mesenchymal transition (EMT), and it has been proposed as a molecular target for breast cancer therapy [6–8]. Although BCL6 expression has been demonstrated to increase in high grade of ductal invasive breast carcinoma patients, many aspects of the molecular mechanisms linked to its expression remain to be elucidated. In this study, we have demonstrated the presence of BCL6 translocation in human breast G3 stage invasive carcinoma samples as compared to G1/G2 ones. Moreover, we have also shown a significant increase of Bcl6 expression by RT-PCR and immunohistochemistry analysis. Our data are aligned with a recent report showing that Bcl6 is upregulated in breast cancer and is associated with poor prognosis, including advanced stages and triple negative molecular subtypes [5]. We have shown that the TUNEL assay demonstrates the absence of apoptosis in breast cancer cells. Moreover, after caspase-3 immunohistochemistry staining, in accordance with the results of TUNEL assay, a few stained cells were recognizable in breast cancer specimens overlapping control values. These results have been confirmed by RT-PCR analysis. We have also studied p53 expression in breast cancer specimens, since BCL6 is a gene promoter involved in p53 protein regulation in terms of suppression of its expression [9]. Immunohistochemistry and RT-PCR demonstrate a significant reduction of the expression of p53 in G3 breast cancer samples as compared to G1/G2 specimens. We also found the increase of P53 mutation percentage in G3 samples respect to G1 and G2 and only one G3 sample with a mutation in the DNA binding site. Moreover, the BCL6 translocation frequency and the p53 positivity in all the patients negatively correlate one to each other. The mutational study of P53 promoter did not revealed any mutation in the Bcl6 binding sites.

The observed heterogeneity in breast cancer can be attributed in part to several cellular components of the inflammatory infiltrate surrounding tumor cells, including macrophages and mast cells, which are both involved in the promotion of angiogenesis occurring during tumor progression in breast cancer. Different literature evidences have shown a close relationship between angiogenesis and tumor progression in breast cancer. Increased vascularity has been shown in mammary ductal carcinoma in situ [10, 11]. Highly vascular tumors have an increased risk of metastasis and a poorer prognosis [12, 13]. Lee et al. [14] have demonstrated that Vascular endothelial growth factor (VEGF) mRNA and protein were significantly more expressed in invasive ductal than in invasive lobular carcinoma. VEGF predicts local relapse and survival in radiotherapy-treated node-negative breast cancer [15]. Increased VEGF expression has been linked to a poorer response to systemic treatments and radiotherapy [16]. The elevated expression of VEGF in breast cancer has been associated to inactivation of tumor suppressor p53 and p53 is likely to be involved in regulating VEGF expression [17, 18]. The identification of VEGF receptors (VEGFRs) on tumor cells themselves revealed the presence of pro-tumorigenic effects of VEGF through the autocrine signaling pathway proliferation, tumor cell survival by protection from apoptosis, cell adhesion and migration, and invasion [19, 20]. Tumor that exhibit overexpression of HER2 also overexpress VEGF and unfavorable prognosis of untreated HER2-positive patients has been linked to an increased angiogenesis [21].

Tumor-associated macrophages in neoplastic lesions are divided in two major subsets: M1 subset, involved in antitumor immunity and anti-angiogenesis and M2 subset which have the opposing roles of enhancing immunosuppression and angiogenesis in tumor progression. Macrophages and mast cells have also been demonstrated to have a role in enhancing angiogenesis in cancer through the release of pro-angiogenic factors and through a complex cross-talk within the tumor microenvironment [22]. In breast cancer, macrophages are to 50% of the tumor mass, and correlate with increased tumor necrosis, angiogenesis, VEGF and VEGFR expression and decreased survival in invasive carcinoma of the breast [23–27]. Moreover, macrophages correlate with hormone receptor negativity, high proliferative activity, high levels of CD8-positive T cells, microvessel density, and survival [28]. In this study, we have demonstrated that total macrophages CD68^+^ and the M2 subpopulation CD163^+^ in interstitial and periglandular position increase in G3 specimens as compared to G1/G2 and control samples. Moreover, CD163^+^ macrophages involved in the angiogenic response and immunosuppression are more numerous as compared to M1 subset, confirming their pro-tumorigenic role in breast cancer.

As concerns mast cells infiltration in breast cancer, we have previously demonstrated in a series of 88 primary female breast cancer that the number and the area occupied by tryptase-positive mast cells, microvascular density and endothelial area, correlate to each other [4]. We have also shown that microvessel counts increase in parallel with the number of tryptase-positive mast cells and their values were significantly higher in human breast cancer sentinel lymph nodes with micrometastases compared with those without [29]. More recently, we have demonstrated that serum tryptase levels may play a role as a novel surrogate angiogenic marker predictive of response to radical surgery in breast cancer patients [30]. In this study, we have shown that tryptase-positive mast cells in periglandular position are more numerous in G3 tumor specimens as compared to G1/G2 and control samples. As concerns microvascular density, we have demonstrated an increase in G1/G2 samples in both interstitial and periglandular positions as compared to G3 and control specimens. Finally, we have demonstrated a negative correlation between BCL6 translocation and p53 positivity and we did not find any BCL6 binding sites mutations on p53 promoter, confirming that Bcl6 is involved in p53 protein regulation in terms of suppression of its expression.

Overall, these data confirm the important role played by epigenetic events, including BCL6 translocation, p53 expression, and that microenvironment components, including macrophage and mast cell infiltration and microvascular density are involved in the regulation of breast cancer progression. Moreover, it has been also demonstrated that the decrease of p53 expression enhances hypoxia-induced hypoxia inducible factor-1 alpha (HIF-1α) levels and increases HIF-1-dependent expression of VEGF in tumor cells. This evidences let us to hypothesize that the downregulation of p53 in our samples could contribute to angiogenic switch activation in response to hypoxia condition during the tumor growth [31]. In this context, the data further validate the notion that a wide range of pathways are involved in cancer development.

## MATERIALS AND METHODS

Primary human breast tumors were retrospectively selected from the archive of the Section of Pathology of the National Cancer Institute “Giovanni Paolo II”, Bari, Italy and the Section of Pathology of the Hospital San Paolo, Bari, Italy. Full ethical approval and signed informed consent from individual patients were obtained to conduct the study. All procedures followed were in accordance with the ethical standards of the responsible committee on human experimentation (institutional and national) and with the Helsinki Declaration of 1964 and later versions. All primary tumors were obtained from patients who had undergone breast cancer surgery. Patients had no received neo-adjuvant therapies and there were subjected to a modified radical mastectomy or to a quadrantectomy. Tumor type and stage were determined according to the World Health Organization classification [32] using conventional histology. The clinicopathological characteristics of the patients were reported in (Table 1).

Bioptic tumor specimens were collected from 30 ductal invasive carcinomas grade 1 (G1), 20 grade 2 (G2), 20 grade 3 (G3), and 15 healthy tissues, histologically confirmed, from patients who undergone breast cancer surgery. Tissues were collected at the time of export of the tumor, fixed in 10% buffered formalin for 24 hours and paraffin-embedded.

### Bcl6, p53, caspase 3, CD31, CD68, CD163, and tryptase immunohistochemistry

Histological sections of 4 μm thickness, collected on poly- L-lysine-coated slides (Sigma Chemical, St Louis, MO, USA), were deparaffinized. The sections were rehydrated in a xylene-graded alcohol scale and then rinsed for 10 minutes in 0.1M PBS. Sections were pre-treated with sodium citrate pH 6.1 or pH 9 (Bcl6) (Dako Corporation, Milan, Italy) in Dako PT Link for antigen retrieval solution for 30 minutes at 98°C and then incubated with rabbit polyclonal anti-CD31 (ab28364, Abcam, Cambridge, UK), mouse monoclonal anti-tryptase (NB-100-64820, Novus Biologicals, Littleton, CO, USA), mouse monoclonal anti-bcl6 (M7211, Dako Corporation, Milan, Italy), rabbit polyclonal anti-caspase3 (NB-600-1235, Novus Biologicals, Littleton, CO, USA), mouse monoclonal anti-CD68 (NCL-CD68-KP1, Novocastra Laboratories Ltd Newcastle, United Kingdom), mouse monoclonal anti-p53 (M7001, Dako Corporation, Milan, Italy)), mouse monoclonal anti-CD163 (NCL-L-CD163, Novocastra Laboratories Ltd Newcastle, United Kingdom), diluted 1:60, 1:1000, 1:150, 1:100, 1:50, 1:30 and 1:200 respectively. Thereafter, the sections were counterstained with Mayer hematoxylin and mounted in synthetic medium. Specific preimmune serum (Dako), replacing the primary antibodies, served as negative control. Sections from each experimental group (n.10), 10 cases per group, were scanned using the whole-slide morphometric analysis scanning platform Aperio Scanscope CS (Leica Biosystems, Nussloch, Germany). All the slides were scanned at the maximum available magnification (40×) and stored as digital high resolution images on the workstation associated with the instrument. Digital slides were inspected with Aperio ImageScope v.11 software (Leica Biosystems, Nussloch, Germany) at 20× magnification and ten fields with an equal area were selected for the analysis at 40× magnification. The protein expression was assessed with the Positive Pixel Count algorithm embedded in the Aperio ImageScope software and reported as positivity percentage, defined as the number of positively stained pixels on the total pixels in the image. The statistical significance of differences between the mean values of the percent labeled areas between tumor breast specimens and control tissues was determined by the 2way Anova test in GraphPad Prism 5.0 software (GraphPad software, La Jolla, CA, USA). Findings were considered significant at P values <0.05.

### Terminal deoxynucleotidyltransferase-mediated dUTP nick end labeling assay-TUNEL test

DNA cleavage was assessed by enzymatic end-labeling of DNA strand breaks using a In Situ Cell Death Detection Kit (Roche, Penzberg, Germany) according to the manufacturer's protocol. Briefly, deparaffinized slides were washed in phosphate-buffered saline (PBS) and permeabilized with 0.1% Triton X-100 and 0.1% sodium citrate for 2 min at 41°C; after rinsing, slides were incubated with 50 ml of terminal deoxynucleotidyltransferase (TdT)- mediated dUTP nick end labeling (TUNEL) reaction mixture, containing TdT- and FITC-labeled dUTP, in a humidified atmosphere for 1 h at 37°C in the dark. Afterwards, slides were rinsed and mounted in antifade solution and images were captured as above with a FITC (TUNEL) filter. The percentages of apoptotic human cells in tumoral sections were evaluated by morphometric analysis carried out on 10 cross reactions from each experimental group, 5 cases per group, using Image Analysis Software (Olympus Italia).

### Real-Time PCR

Total RNA was extracted from FFPE tissue blocks using the RecoverAll^TM^ Total Nucleic Acid isolation kit (Ambion, Life Technologies, Inc., Austin, TX, USA) and then used to synthesize the first-strand c-DNA with the IScriptcDNA Synthesis kit (Bio-Rad Laboratories, Hercules, CA, USA), according to the manufacturer's instructions. To detect Bcl-6, p53, caspase 3 expression, cDNA was amplified with the TaqMan Gene Expression Master Mix (Applied Biosystems, Foster City, USA). PCR amplification was performed using the Chromo4 Real-time PCR Detection System (Bio-Rad Laboratories). Samples were normalized to human B2M (β2 microglobulin). Table 2 shows the sequences of the taqman probes (Applied Biosystems, Life technologies) used for Bcl-6, p53, caspase 3 amplification, respectively.

**Table 2:** TaqMan gene expression assays for real-time PCR

B2M	Hs99999907_m1
BCL6	Hs00153368_m1
CASP3	Hs00234385_m1
TP53	Hs01034253_m1

### Fish analysis

Interphase FISH was performed on thin sections of formalin-fixed paraffin-embedded (FFPE) tumor samples. All cases were screened for BCL6 translocation with a Vysis LSI BCL6 dual-color break-apart probe (Abbott Molecular Abbott Park, Illinois, USA). In brief, 4-μm paraffin-embedded sections, mounted on coated slides, were deparaffinized and rehydrated. Slides were next incubated at 96°C in Tris/EDTA acid buffer solution for 15 minutes, washed in sterile water and treated in 0.01 N HCL solution at 37°C for 2 minutes. Enzymatic digestion was then induced by adding 200 μl 0.4% pepsin (Sigma-Aldrich, St. Louis, MO, USA) solution and incubating at 37°C for 15 minutes. Thereafter, tissue samples were washed with sterile water, dehydrated in ethanol, and air dried. The Bcl6 dual-color break-apart probe was used for hybridization according to the manufacturer's protocol. Probe and target were co-denatured at 73°C for 5 minutes, followed by overnight hybridization at 37°C on a StatSpinThermoBrite (Abbott Molecular, Abbott Park, Illinois, USA). Post-hybridization washing was carried out at 72 ± 1°C in 0.4XSSC/0.3%NP40 for 2 minutes and in 2XSSC/0.1%NP40 at room temperature for 1 minute. Then the slides were counterstained and mounted with DAPI-Antifade (Cytocell).

The interphase FISH analysis was performed using an Olympus BX51 fluorescence microscope (Olympus Italia, Rozzano, Italy) with 100X objective lenses and a triple band-pass filter for FITC, Texas Red and DAPI. Images were captured using a high-resolution digital camera (DP70, Olympus Italia) transmitting image data to a PC equipped with appropriate software for images acquisition and analysis (AnalySIS, Olympus Italia). Hybridization signals were counted in 200 morphologically intact nuclei for each sample. Signals were considered co-localized when the distance between them was equal to or smaller than the size of one hybridization signal. Only co-localized green and red signals, often resulting in a yellow signal, were considered representative of intact BCL6 loci. By contrast, a BCL6 translocation breakpoint is easily recognized by the presence of scattered single red and green signals.

### Mutation screening of P53

Genomic DNA was extracted from tumor sections using the QIAamp Mini Kit (Qiagen, Hilden, Germany), according to the manufacturer's instructions, and quantified on a BioSpectrometer Plus (Eppendorf, Hamburg, Germany). The entire coding (exons 2-11) and promoter regions of P53, were amplified by standard PCR protocols using Taq DNA Polymerase (Thermo Scientific, USA) and the primer pairs with sequences available on request. Direct sequencing was performed using the BigDye Terminator v1.1 Cycle Sequencing Kit (Applied Biosystems) according to the manufacturer's instructions on an ABI 310 Genetic Analyzer (Applied Biosystems). The sequence analysis software Alamut® (Interactive Biosoftware) was used to interpret variants.

### TCGA data

Data related to the mutational status of P53 and BCL6 in invasive breast carcinoma worldwide are also been acquired by the Cancer Genome Atlas (TCGA) consultation at the portal https://portal.gdc.cancer.gov. The TCGA contains genomic, epigenomic and proteomic data from more than 10,000 samples derived from 33 types of cancer providing an important opportunity for the discovery new of signal pathways key regulators or new functions of preexisting members in pathways [33].

### Statistical analysis

Data related to the three experimental groups, breast control tissue, G1 and G2 breast tumors together and G3 tumor samples are reported as means ± SEM. Newman-Keuls multiple comparisons post-test was used to compare all treatment groups after one-way ANOVA. The Graph Pad Prism 5.0 statistical package (GraphPad Software, San Diego, CA, USA) was used for analyses and the limit for statistical significance was set at P<0.05. Correlation analysis between CD68 and CD31, CD163 and CD31, tryptase and CD31, Bcl6 and CD31, Bcl6 translocation and p53 positivity was performed with the Spearman non parametric correlation test. The Graph Pad Prism 5.0 statistical package (GraphPad Software, San Diego, CA, USA) was used for analyses and the limit for statistical significance was set at P<0.05.

## Abbreviations

G1: Ductal invasive carcinomas grade 1
G2: Ductal invasive carcinomas grade 2
G3: Ductal invasive carcinomas grade 3
CTRL: Control
p53: phosphoprotein 53
CD31: cluster of differentiation 31
CD68: cluster of differentiation 68
CD163: cluster of differentiation 163
Bcl6: B-cell lymphoma 6 protein
BCL6: B-cell lymphoma 6 gene
